# Correction: Natural epigenetic polymorphisms lead to intraspecific variation in Arabidopsis gene imprinting

**DOI:** 10.7554/eLife.08658

**Published:** 2015-05-18

**Authors:** Daniela Pignatta, Robert M Erdmann, Elias Scheer, Colette L Picard, George W Bell, Mary Gehring

Pignatta D, Erdmann RM, Scheer E, Picard CL, Bell GW, Gehring M. 2014. Natural epigenetic polymorphisms lead to intraspecific variation in Arabidopsis gene imprinting. *eLife* **3**:e03198. doi: 10.7554/eLife.03198. Published 3 July 2014

Recently, a reader noticed that some lines in Figure 1—source data 5 did not match the corresponding data in Figure 1—source data 3. We discovered that when we were preparing Figure 1—source data 5 we transposed some lines of data from Figure 1—source data 3. Thus, because of the transposition, some p-values in Figure 1—source data 5 were calculated incorrectly (a side-by-side comparison is shown in Supplementary file 1, file held on figshare under http://dx.doi.org/10.6084/m9.figshare.1409445). We have now corrected all errors in Figure 1—source data 5. The correction does not change any of the conclusions that we drew from these data, which were ‘Imprinting calls based on whole-genome mRNA-seq were validated by sequencing or performing CAPs digestion on RT-PCR amplicons of 29 genes from independently isolated embryo and endosperm RNA samples (Figure 1—figure supplement 2, Figure 1—source data 5); results were mostly consistent with the mRNA-seq data’. We apologize for the mistake.

